# St. John's Wort Has Metabolically Favorable Effects on Adipocytes *In Vivo*

**DOI:** 10.1155/2014/862575

**Published:** 2014-05-20

**Authors:** Scott Fuller, Allison J. Richard, David M. Ribnicky, Robbie Beyl, Randall Mynatt, Jacqueline M. Stephens

**Affiliations:** ^1^Pennington Biomedical Research Center, Louisiana State University, 6400 Perkins Road, Baton Rouge, LA 70808, USA; ^2^Biotech Center, Rutgers University, New Brunswick, NJ 08901, USA

## Abstract

In addition to serving as a storage site for reserve energy, adipocytes play a critical role in whole-body insulin sensitivity and glucose metabolism. St. John's Wort (SJW) is a botanical supplement widely used as an over-the-counter treatment of depression and a variety of other conditions associated with anxiety and nerve pain. Previous studies in our laboratory demonstrated that SJW inhibits insulin-stimulated glucose uptake and adipocyte differentiation in cultured murine and mature human adipocytes. To investigate the effects of SJW on adipocyte function *in vivo*, we utilized C57BL/6J mice. In our studies, mice were administered SJW extract (200 mg/kg) once daily by gavage for two weeks. In contrast to our *in vitro* studies, mice treated with SJW extract showed increased levels of adiponectin in white adipose tissue in a depot specific manner (*P* < 0.01). SJW also exerted an insulin-sensitizing effect as indicated by a significant increase in insulin-stimulated Akt serine phosphorylation in epididymal white adipose tissue (*P* < 0.01). Food intake, body weight, fasting blood glucose, and fasting insulin did not differ between the two groups. These results are important as they indicate that SJW does not promote metabolic dysfunction in adipose tissue *in vivo*.

## 1. Introduction 

Nutritional supplements derived from botanical sources have a long history of human use in promoting general health and in the treatment of disease. While there is considerable empirical evidence supporting the use of botanicals as adjuncts to overall sound nutrition, our understanding of the molecular mechanisms of action of botanicals remains incomplete. Additionally, some plant-based supplements have been associated with negative side effects for certain subsets of the human population. Therefore scientific investigations aimed at evaluating the safety, efficacy, and molecular mechanisms by which botanicals exert their biological effects have intensified.

The physiological importance of adipocytes as lipid storage depots has long been recognized. However, since the discovery of the adipose-derived hormone leptin in 1995 [1], an accumulating body of evidence has established that adipose tissue exerts a broader range of effects throughout the organism than had been previously understood. Some of these effects include the regulation of whole-body insulin sensitivity, glucose metabolism, and feeding behavior [2, 3]. Obesity is the primary pathology of adipocytes and is implicated in the etiology of type 2 diabetes mellitus (T2DM), cardiovascular disease, and some types of cancer. The link between obesity and other diseases suggests that inhibiting the growth of adipose tissue could be a promising therapeutic target. However, evidence has emerged indicating that decreased adipocyte differentiation and loss of adipose tissue expansion result in adverse metabolic consequences, including insulin resistance and T2DM [4, 5]. In view of the importance of adipocytes in maintaining proper metabolic function, it is possible that any botanical product that modulates adipocytes could affect the metabolic status of the entire organism.

Previous work in our laboratory characterized the effects of St. John's Wort (*Hypericum perforatum L.*) on adipocytes in cell culture. These studies demonstrated that leaf and flower extracts from St. John's Wort (SJW) inhibited adipocyte differentiation in 3T3-L1 cells. Moreover, SJW induced insulin resistance and blunted insulin-stimulated glucose uptake in both murine and human adipocytes* in vitro* [6, 7]. This line of experimental evidence raised questions about potential adverse metabolic consequences associated with the use of SJW as a botanical supplement. SJW is widely available over the counter and is used primarily to treat depression, along with other conditions [8, 9]. Considering the widespread use and availability of SJW and the disease burden associated with the obesity and diabetes epidemics, we sought to determine whether the deleterious metabolic effects of SJW extracts observed in cell culture could occur in an* in vivo *experimental model. To address this question we used C57BL/6J mice supplemented with SJW extract and measured biological markers of metabolic status relevant to insulin signaling and glucose uptake.

## 2. Materials and Methods

### 2.1. Animals

All animal experiments were approved by the Institutional Animal Care and Use Committee at the Pennington Biomedical Research Center. 12-week-old C57BL/6J male mice fed a chow diet were purchased from Jackson Laboratory (Bar Harbor, ME). The mice were housed individually under constant temperature and humidity (21 ± 2°C with humidity 65–75%) and a 12 : 12 h light-dark cycle. Mice were allowed access to food and water* ad libitum* for the duration of the study.

### 2.2. Experimental Design and Diet

After exit from quarantine, mice were weighed and body composition was measured by NMR. All mice were gavaged once daily for 6 days with 5% DMSO/PBS. Following the gavage lead-in, mice were weighed and body composition was measured, and then the mice were randomized into two groups (13 mice per group) based on body weight. The control group was gavaged daily with 5% DMSO/PBS for two weeks. The treatment group was gavaged daily with 200 mg/kg SJW extract in 5% DMSO/PBS for two weeks. SJW extract was prepared and provided by the Rutgers Botanical Core Facility [7]. All mice were fed a standard chow diet for the duration of the study. Food intake and body weight were recorded daily. Body composition was determined by NMR at baseline, week 1, and week 2 of the study. At the end of the study, mice were fasted overnight and given an intraperitoneal (IP) injection of saline or insulin (0.02 U). At 10 minutes after IP injection, the animals were euthanized. White adipose tissue (WAT) from the epididymal and inguinal depots was immediately dissected, placed in liquid nitrogen, and then stored at −80°C for future analysis.

### 2.3. Blood Glucose and Insulin Analyses

Submandibular blood was collected at baseline, week 1, and week 2 following a four-hour fast. Plasma fasting glucose levels were measured using a YSI glucose analyzer (YSI Life Sciences, Yellow Springs, OH). Fasting insulin levels were determined by a Crystal Chem rat/mouse insulin enzyme-linked immunosorbent (ELISA) kit (Crystal Chem, Downers Grove, IL).

### 2.4. Determination of Homeostasis Model Assessment of Insulin Resistance

The homeostasis model assessment of insulin resistance (HOMA-IR) was calculated using glucose and insulin concentrations obtained after a 4-hour fast, using the following formula: fasting  glucose  (mg/dL) × fasting  insulin  (*μ*U/mL)/405 [10].

### 2.5. Gel Electrophoresis and Immunoblotting

Tissue lysates were prepared from WAT by homogenization in an ice-cold nondenaturing buffer containing 150 mM NaCl, 10 mM Tris, pH 7.4, 1 mM EGTA, 1 mM EDTA, 1% Triton X-100, 0.5% Igepal CA-630, 1 *μ*M PMSF, 1 *μ*M pepstatin, 50 trypsin inhibitory milliunits of aprotinin, 10 *μ*M leupeptin, 1 mM 1,10-phenanthroline, and 0.2 mM sodium vanadate. The homogenates were then centrifuged at 13,000 ×g for 10 minutes at 4°C. The floating lipid layer was removed, and the protein concentrations of the supernatants were determined by a BCA kit (Thermo Scientific, Rockford, IL) according to the manufacturer's instructions. Proteins (50 *μ*g) were then separated on 10% polyacrylamide (acrylamide from National Diagnostics) gels containing SDS according to the method of Laemmli [11]. For the determination of high molecular weight adiponectin, proteins (10 *μ*g) were separated on 5% nondenaturing gels. Following gel electrophoresis the proteins were transferred to nitrocellulose membranes in transfer buffer containing 25 mM Tris, 192 mM glycine, and 20% methanol. Membranes were then blocked in casein blocking buffer (Licor Biosciences, Lincoln, NE) for 1 hour at room temperature before being subjected to western blotting. Protein abundance was detected by antibodies against adiponectin (Thermo Scientific number MA1-054, Rockford, IL), Akt, and phosphorylated Akt^(Ser473)^ (Cell Signaling number 2920 and number 9271, Danvers, MA). Membranes were then incubated in light-protective black boxes for one hour at room temperature with the appropriate anti-mouse or anti-rabbit IgG IRDye-conjugated secondary antibodies (Licor Biosciences, Lincoln, NE) and scanned with the Odyssey infrared scanner (Licor Biosciences, Lincoln, NE). Optical densities of all protein bands were analyzed using Image Studio Lite software (Licor Biosciences, Lincoln, NE).

### 2.6. Statistical Analysis

Statistical analyses for body weight, food intake, fasting glucose, and fasting insulin were performed using the mixed procedure in SAS version 9.3 (SAS Institute, Cary, NC). GraphPad Prism software (GraphPad Software Inc., La Jolla, CA) was used to compare the optical densities of adiponectin. For adiponectin, data was analyzed by an independent two-sample *t*-test. Akt and Aktp^(Ser473)^ were compared using least square means from a linear model. Tests were considered as significant at *P* values of <0.05.

## 3. Results

To examine the effects of SJW* in vivo*, we performed a gavage lead-in for six days so that mice were accustomed to being handled and gavaged daily. After the lead-in, the mice were weighed and body composition was measured, and then the mice were randomized into two groups based on body weight. The control group was gavaged daily with 5% DMSO/PBS for two weeks. The treatment group was gavaged daily with 200 mg/kg SJW extract in 5% DMSO/PBS for two weeks. At the end of the study, animals were sacrificed and white adipose tissue (WAT) from the epididymal and inguinal depots was removed and stored for future analysis.

**Figure 1:**
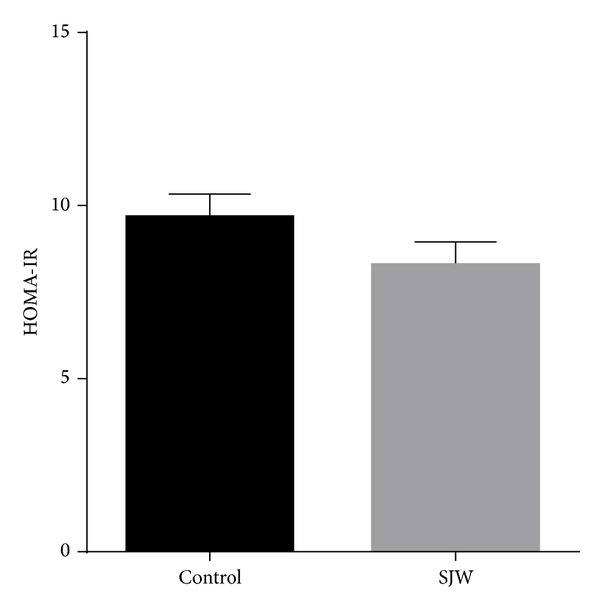
SJW does not alter HOMA-IR in mice. C57BL/6J mice were gavaged daily for two weeks with SJW extract. HOMA-IR was calculated from fasting insulin and glucose levels. Data are presented as mean ± SEM (*n* = 13), *P* = 0.28.

The effects of SJW on food intake, body weight, fasting blood glucose, and fasting insulin were assessed (Table 1). HOMA-IR was calculated and did not differ significantly between the SJW-treated mice and controls at week 2 (Figure 1). Body weight and food intake also were not different between the SJW-treated mice and controls at baseline or at week 2. Fasting blood glucose decreased in both the SJW and control groups over the two-week duration of the study, but the difference was not statistically significant. Fasting blood glucose was slightly lower in the SJW group at both baseline and week 2 compared to the control group, but this difference was also not significant. Over the 2-week time course of the study, the fasting insulin levels of the SJW group decreased while the levels of the control group increased, although these differences did not reach statistical significance.

**Figure 2:**
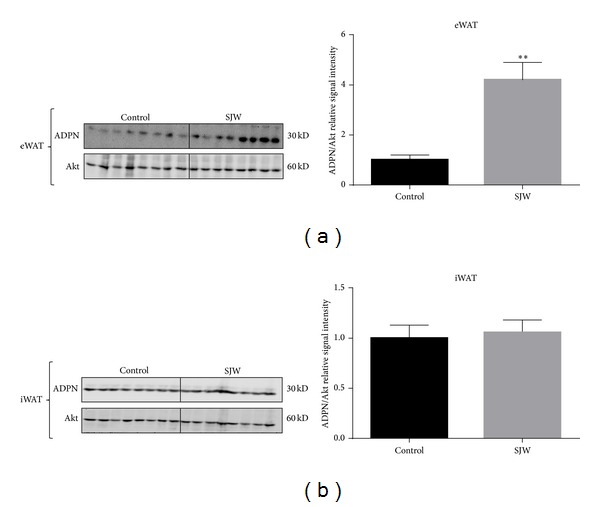
SJW increases the levels of adiponectin in WAT in a depot specific manner. WAT from the epididymal (a) and inguinal (b) depots was removed and immediately frozen in liquid nitrogen. Tissue extracts were subjected to western blot analysis. There were 13 mice per condition and, of those, 8 mice per condition are shown. Bar graphs represent relative optical densities of adiponectin, normalized to total Akt derived from the same gel. *N* = 8/group. **P < 0.01 versus control. Data are presented as means ± SEM.

**Figure 3:**
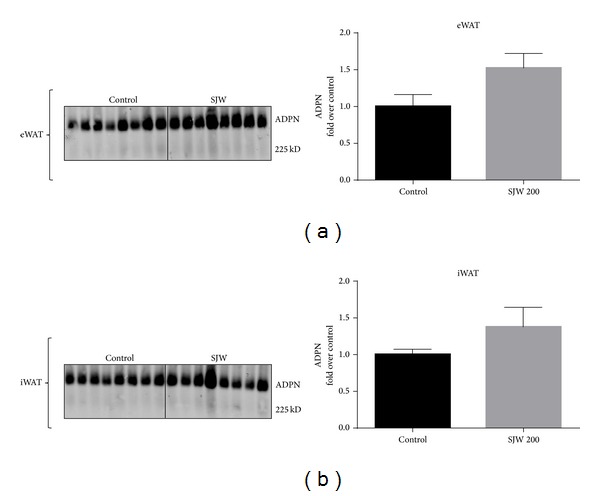
SJW increases high molecular weight (HMW) adiponectin in epididymal (eWAT) and inguinal (iWAT) fat depots. Proteins from eWAT and iWAT were separated by native PAGE and visualized by western blot analysis. Bar graphs represent relative optical densities of HMW adiponectin, normalized to those in the control group. *N* = 8/group. Data are presented as means ± SEM.

**Figure 4:**
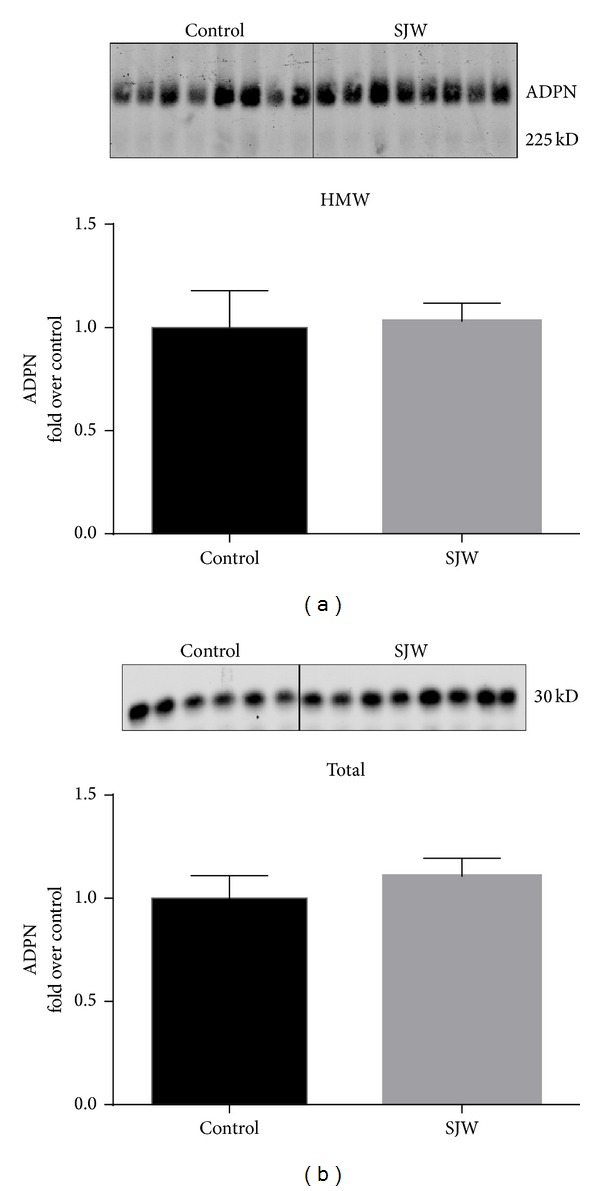
SJW does not affect the serum levels of high molecular weight (a) and total adiponectin (b) in chow-fed C57BL/6J mice. Serum proteins were separated by native PAGE and SDS-PAGE (resp., for HMW and total adiponectin (ADPN) content) and visualized by western blot analysis. Densitometric analysis is shown below each blot. Data are means ± SEM. *N* = 8/group for HMW ADPN. For total ADPN, *N* = 6 for controls and *N* = 8 for SJW.

To determine if SJW had effect on adipocytes, we examined the expression of adiponectin. Adiponectin is a fat specific hormone that is associated with insulin sensitivity and metabolic health. As shown in Figure 2(a), our analysis showed a significant (*P* < 0.01) increase in total adiponectin in the epididymal white adipose depot of the SJW-treated mice compared to the control mice. However, total adiponectin levels in inguinal WAT did not show a difference between the two groups (Figure 2(b)). High molecular weight adiponectin is associated with decreased risks of diabetes and metabolic health. We used nondenaturing gels to examine the levels of high molecular weight adiponectin in the epididymal and inguinal WAT depots. As shown in Figure 3(a), there was an increase in the levels of HMW adiponectin in SJW-treated mice. The increase was approximately 1.5-fold over control animals and approached but did not reach statistical significance (*P* = 0.068). In inguinal WAT we observed a nonsignificant (*P* = 0.21) increase in the level of HMW adiponectin in the SJW-treated animals compared to the controls (Figure 3(b)). The effect of SJW on adiponectin levels in the serum was also assessed. Following a two-week SJW treatment, there were no differences in the levels of HMW or total adiponectin in the serum (Figure 4).

**Figure 5:**
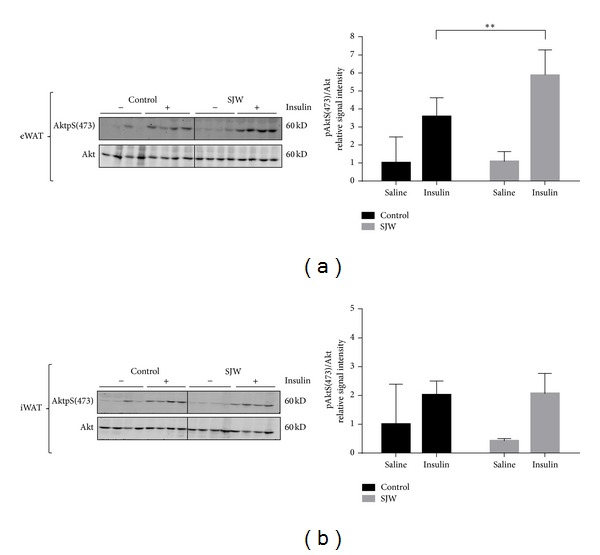
SJW modulates insulin sensitivity in WAT in a depot specific manner. Prior to euthanasia the animals were fasted overnight and administered an IP injection of insulin or saline for 10 minutes. WAT from epididymal (a) and inguinal (b) fat depots were removed and immediately frozen in liquid nitrogen. Tissue extracts were subjected to western blot analysis. There were 6 mice per condition and, of those, 4 mice per condition are shown. Bar graphs represent relative optical densities of AktpS(473), normalized to the respective total Akt band intensities derived from the same gel. *N* = 4/group. **P < 0.01. Data are presented as means ± SEM.

To study the effect of SJW on insulin signaling in adipose tissue, we examined the levels of Akt phosphorylation in adipose tissue of the mice in response to an acute intraperitoneal (IP) insulin injection. Adipose tissue from the epididymal and inguinal fat depots was collected from control mice or SJW-treated mice that were euthanized 10 minutes after an IP injection of either insulin or saline. Insulin sensitivity was assessed by examining the serine phosphorylation of Akt, a well-established indicator of insulin signaling. The levels of Akt phosphorylation at serine^473^ were normalized to total Akt levels. Our results indicate that SJW modulates insulin sensitivity in WAT in a depot specific manner. As shown in Figure 5(a), insulin-stimulated phosphorylation of Akt in eWAT was significantly increased in SJW-treated mice compared to controls (*P* < 0.01). In the inguinal fat depot, there was no increase in insulin-stimulated Akt phosphorylation (Figure 5(b)).

## 4. Discussion

Previous* in vitro* studies in our laboratory demonstrated that SJW inhibited adipogenesis, induced insulin resistance, and inhibited insulin-stimulated glucose uptake in mouse and human fat cells [6, 7]. On the basis of these observations, we hypothesized that SJW might exert similar effects* in vivo* and present a risk to metabolically compromised individuals that use this botanical supplement. Hence, we studied the effects of SJW supplementation on insulin sensitivity in adipose tissue and the production of the adipocyte specific hormone, adiponectin. Our results indicate that SJW exerts differential and metabolically favorable effects in adipose tissue.

The most notable finding of our current study is that SJW increased adiponectin levels in WAT in a depot specific manner. Prior experiments in our laboratory using cultured murine and human adipocytes showed that SJW potently suppressed adiponectin levels [6, 7]. However, in the present study, we observed a statistically significant increase in adiponectin in the eWAT of the mice treated with SJW. Since its discovery in 1995 [12], adiponectin has been studied extensively. Among the biological effects attributed to this adipocyte-derived hormone are increased insulin sensitivity, enhanced glucose uptake, modulation of endothelial function, and antiatherogenesis and anti-inflammatory activity [13–18]. Although we discovered a statistically significant increase in adiponectin level only in the eWAT of the SJW-treated mice, this finding warrants particular attention in at least two aspects. First, considering the well-established biological effects of adiponectin and the results of our earlier work showing that SJW decreased adiponectin, the unexpected finding that treatment with SJW resulted in a significant increase in adiponectin in the eWAT depot suggests that in the whole animal there are unlikely to be negative metabolic consequences of SJW supplementation. Although SJW treatment resulted in a significant increase in adiponectin in epididymal fat only, we hypothesize that an extended treatment of SJW would likely increase adiponectin levels in other adipose tissues and in circulation. Overall, these results demonstrate that SJW supplementation does not have adverse effects on metabolic status* in vivo*, as assessed by adiponectin expression.

In agreement with the results of our findings on adiponectin, we also observed an increase in the insulin-stimulated phosphorylation of Akt in the eWAT of SJW-treated mice compared with controls (*P* < 0.01). Akt plays a central role in several critical cellular responses, including cell growth and survival, protein synthesis, angiogenesis, and glycogen synthesis [19]. The involvement of Akt in insulin signaling [20, 21] is of particular relevance in our study. The observation that SJW resulted in an increase in insulin-stimulated phosphorylation of Akt on serine 473 is an indicator of enhanced insulin sensitivity. Impaired insulin sensitivity is a classic symptom of metabolic syndrome and the finding that both adiponectin and Akt phosphorylation increased in a depot specific manner in the WAT of SJW-treated animals provides evidence of an insulin-sensitizing effect of this botanical extract. Of note, these results are divergent from our previous* in vitro* studies [6, 7]. However, these* in vivo* studies are more translational to humans and the likely negative effects of SJW observed* in vitro* could be due to lack of metabolism of SJW extracts in the cell culture model system.

We also report that body weight and food intake did not differ with SJW treatments. These results are expected since the mice were maintained on a chow diet that was not designed to evoke profound changes in these parameters, particularly over a 2-week time course. However, both fasting plasma glucose and insulin decreased over the 2-week duration of SJW treatment, whereas in the controls we observed a decrease in glucose and an increase in fasting insulin. We also note that the HOMA-IR of the SJW-treated mice was somewhat lower than in controls, although this difference did not reach statistical significance. While we recognize that these changes were not statistically significant, the decrease in fasting insulin in all of the SJW-treated mice with an accompanying modest decrease in blood glucose is consistent with the increased adiponectin and enhanced insulin sensitivity in adipose tissue that we observed.

The present study does not allow conclusions to be drawn about the specific bioactive compounds in SJW responsible for the insulin-sensitizing effects that we observed in adipose tissue. Hypericin and hyperforin are the two most well-known bioactive agents in SJW. In cultured adipocytes, neither of these bioactives modulates adipocyte differentiation or insulin signaling [7]. However, the antidepressant activities of SJW have been attributed to hyperforin, which is also reported to have anti-inflammatory and antiangiogenic effects [22]. Nonetheless, SJW contains many other bioactive compounds of which approximately 50–70% have been identified; these known phytochemicals belong to a variety of chemical classifications [23]. In addition to hypericin and hyperforin, which represent naphthodianthrones and phloroglucinols, SJW is known to contain numerous flavonoids, biflavonoids, and phenolic acids [24]. Hence, it is also unlikely that either hypericin or hyperforin alone accounts for the beneficial effects of SJW on insulin action in adipose tissue in the present* in vivo* study. Future studies measuring the effects of isolated bioactive constituents of SJW will be required to determine the insulin-sensitizing chemical components in SJW.

SJW is readily available over the counter and is used worldwide as an herbal supplement in the treatment of depression and a variety of other conditions. Considering the availability and widespread use of SJW, efforts to learn about the biological effects of this botanical are worthwhile. SJW has previously been shown to interfere with the action of several drugs and bioactive compounds in SJW have been shown to activate the pregnane X receptor, which activates genes that affect the metabolism and excretion of drugs [25–27]. One factor that might account for the discordance between the results of our cell culture studies compared to the present investigation is that a metabolite of SJW is responsible for the beneficial effects we observed in these studies. Pharmacokinetic studies can differ in their assessments of the bioavailability of compounds when administered* in vivo* [28–30]. We cannot exclude the possibility that absorption and bioavailability of the active ingredients in the SJW extracts influenced the metabolic effects we observed. We also note that the dose (200 mg/kg) of SJW extract selected is similar to that administered in other murine models that studied the effects of SJW on anxiety and gallstone disease [31, 32]. Our experimental protocol utilized gavage in order to overcome any possible food aversion and to ensure that a precise dosage of the SJW extract was administered to each animal in the treatment group. Since the duration of our study was two weeks, extrapolating the positive metabolic effects we observed should be approached with caution. Additionally, it is possible that in the context of a high fat diet (Western diet) SJW may not exhibit the same beneficial metabolic effects.

## 5. Concluding Remarks

To our knowledge, this is the first study to specifically evaluate the effects of SJW on adipose tissue in a murine model. Our novel findings indicate that SJW treatment significantly increased adiponectin levels in the eWAT of C57BL/6J mice maintained on a chow diet. These results are divergent from our* in vitro* studies on mouse and human adipocytes [6, 7]. Overall, the results of the present study do not implicate SJW as a cause of metabolic dysfunction in mice and suggest that SJW does not exacerbate metabolic risk in people who use this botanical supplement. In conclusion, our results reveal that a two-week treatment of SJW can enhance adiponectin expression and insulin action in epididymal adipose tissue.

## Figures and Tables

**Table 1:** Data for food intake, body weight, fasting plasma glucose, and fasting plasma insulin measured at baseline and week 2 of the study.

Group	Food intake/day (g)	Body weight (g)		Fasting glucose (mg/dL)		Fasting insulin (pg/mL)	
		Baseline	Week 2	Baseline	Week 2	Baseline	Week 2
Control	3.779 ± 0.74	26.95 ± 0.37	27.68 ± 0.45	161.17 ± 6.54	142.90 ± 5.45	826.5 ± 40.32	956.1 ± 70.59
SJW	3.723 ± 0.62	26.95 ± 0.35	27.32 ± 0.49	158.31 ± 6.43	137.77 ± 4.74	905.5 ± 62.58	840.1 ± 40.75

Values are presented as means ± SEM.

## References

[1] Halaas JL, Gajiwala KS, Maffei M (1995). Weight-reducing effects of the plasma protein encoded by the obese gene. *Science*.

[2] Mantzoros CS (1999). The role of leptin in human obesity and disease: a review of current evidence. *Annals of Internal Medicine*.

[3] Berg AH, Combs TP, Du X, Brownlee M, Scherer PE (2001). The adipocyte-secreted protein Acrp30 enhances hepatic insulin action. *Nature Medicine*.

[4] Danforth E (2000). Failure of adipocyte differentiation causes type II diabetes mellitus?. *Nature Genetics*.

[5] Kim J, van de Wall E, Laplante M (2007). Obesity-associated improvements in metabolic profile through expansion of adipose tissue. *The Journal of Clinical Investigation*.

[6] Amini Z, Boyd B, Doucet J, Ribnicky DM, Stephens JM (2009). St. John’s wort inhibits adipocyte differentiation and induces insulin resistance in adipocytes. *Biochemical and Biophysical Research Communications*.

[7] Richard AJ, Amini ZJ, Ribnicky DM, Stephens JM (2012). St. John’s wort inhibits insulin signaling in murine and human adipocytes. *Biochimica et Biophysica Acta*.

[8] Greeson JM, Sanford B, Monti DA (2001). St. John’s wort (*Hypericum perforatum*): a review of the current pharmacological, toxicological, and clinical literature. *Psychopharmacology*.

[9] Butterweck V (2003). Mechanism of action of St John’s wort in depression: what is known?. *CNS Drugs*.

[10] Matthews DR, Hosker JP, Rudenski AS, Naylor BA, Treacher DF, Turner RC (1985). Homeostasis model assessment: insulin resistance and *β*-cell function from fasting plasma glucose and insulin concentrations in man. *Diabetologia*.

[11] Laemmli UK (1970). Cleavage of structural proteins during the assembly of the head of bacteriophage T4. *Nature*.

[12] Scherer PE, Williams S, Fogliano M, Baldini G, Lodish HF (1995). A novel serum protein similar to C1q, produced exclusively in adipocytes. *The Journal of Biological Chemistry*.

[13] Ouchi N, Kihara S, Arita Y (1999). Novel modulator for endothelial adhesion molecules: adipocyte-derived plasma protein adiponectin. *Circulation*.

[14] Yamauchi T, Kamon J, Waki H (2001). The fat-derived hormone adiponectin reverses insulin resistance associated with both lipoatrophy and obesity. *Nature Medicine*.

[15] Yamauchi T, Kamon J, Minokoshi Y (2002). Adiponectin stimulates glucose utilization and fatty-acid oxidation by activating AMP-activated protein kinase. *Nature Medicine*.

[16] Ouchi N, Kobayashi H, Kihara S (2004). Adiponectin stimulates angiogenesis by promoting cross-talk between AMP-activated protein kinase and Akt signaling in endothelial cells. *The Journal of Biological Chemistry*.

[17] Whitehead JP, Richards AA, Hickman IJ, Macdonald GA, Prins JB (2006). Adiponectin-a key adipokine in the metabolic syndrome. *Diabetes, Obesity and Metabolism*.

[18] Awazawa M, Ueki K, Inabe K (2011). Adiponectin enhances insulin sensitivity by increasing hepatic IRS-2 expression via a macrophage-derived IL-6-dependent pathway. *Cell Metabolism*.

[19] Brazil DP, Hemmings BA (2001). Ten years of protein kinase B signalling: a hard Akt to follow. *Trends in Biochemical Sciences*.

[20] Cross DA, Alessi DR, Cohen P, Andjelkovich M, Hemmings BA (1995). Inhibition of glycogen synthase kinase-3 by insulin mediated by protein kinase B. *Nature*.

[21] Kohn AD, Kovacina KS, Roth RA (1995). Insulin stimulates the kinase activity of RAC-PK, a pleckstrin homology domain containing ser/thr kinase. *EMBO Journal*.

[22] Medina MA, Martínez-Poveda B, Amores-Sánchez MI, Quesada AR (2006). Hyperforin: more than an antidepressant bioactive compound?. *Life Sciences*.

[23] Butterweck V, Schmidt M (2007). St. John’s wort: role of active compounds for its mechanism of action and efficacy. *Wiener Medizinische Wochenschrift*.

[24] Barnes J, Anderson LA, Phillipson JD (2001). St John’s wort (*Hypericum perforatum* L.): a review of its chemistry, pharmacology and clinical properties. *Journal of Pharmacy and Pharmacology*.

[25] Goodwin B, Redinbo MR, Kliewer SA (2002). Regulation of cyp3a gene transcription by the pregnane X receptor. *Annual Review of Pharmacology and Toxicology*.

[26] Kober M, Pohl K, Efferth T (2008). Molecular mechanism underlying St. John’s wort drug interactions. *Current Drug Metabolism*.

[27] Moore LB, Goodwin B, Jones SA (2000). St. John’s wort induces hepatic drug metabolism through activation of the pregnane X receptor. *Proceedings of the National Academy of Sciences of the United States of America*.

[28] Choudhury R, Chowrimootoo G, Srai K, Debnam E, Rice-Evans CA (1999). Interactions of the flavonoid naringenin in the gastrointestinal tract and the influence of glycosylation. *Biochemical and Biophysical Research Communications*.

[29] Felgines C, Texier O, Morand C (2000). Bioavailability of the flavanone naringenin and its glycosides in rats. *The American Journal of Physiology-Gastrointestinal and Liver Physiology*.

[30] Kanaze FI, Bounartzi MI, Georgarakis M, Niopas I (2007). Pharmacokinetics of the citrus flavanone aglycones hesperetin and naringenin after single oral administration in human subjects. *European Journal of Clinical Nutrition*.

[31] He J, Nishida S, Xu M, Makishima M, Xie W (2011). PXR prevents cholesterol gallstone disease by regulating biosynthesis and transport of bile salts. *Gastroenterology*.

[32] Kumar A, Singh A (2007). Protective effect of St. John’s wort (*Hypericum perforatum*) extract on 72-hour sleep deprivation-induced anxiety-like behavior and oxidative damage in mice. *Planta Medica*.

